# Genome sequence of *Pseudomonas aeruginosa* PAO1161, a PAO1 derivative with the ICE*Pae*1161 integrative and conjugative element

**DOI:** 10.1186/s12864-019-6378-6

**Published:** 2020-01-06

**Authors:** Adam Kawalek, Karolina Kotecka, Magdalena Modrzejewska, Jan Gawor, Grazyna Jagura-Burdzy, Aneta Agnieszka Bartosik

**Affiliations:** 1 Institute of Biochemistry and Biophysics, Polish Academy of Sciences, Department of Microbial Biochemistry, Warsaw, Poland; 2 Institute of Biochemistry and Biophysics, Polish Academy of Sciences, DNA Sequencing and Oligonucleotide Synthesis Laboratory, Warsaw, Poland

**Keywords:** *Pseudomonas aeruginosa*, Genome sequence, Integrative conjugative element, Mercury resistance

## Abstract

**Background:**

*Pseudomonas aeruginosa* is a cause of nosocomial infections, especially in patients with cystic fibrosis and burn wounds. PAO1 strain and its derivatives are widely used to study the biology of this bacterium, however recent studies demonstrated differences in the genomes and phenotypes of derivatives from different laboratories.

**Results:**

Here we report the genome sequence of *P. aeruginosa* PAO1161 laboratory strain, a *leu*-, Rif^R^, restriction-modification defective PAO1 derivative, described as the host of IncP-8 plasmid FP2, conferring the resistance to mercury. Comparison of PAO1161 genome with PAO1-UW sequence revealed lack of an inversion of a large genome segment between rRNA operons and 100 nucleotide polymorphisms, short insertions and deletions. These included a change in *leuA*, resulting in E108K substitution, which caused leucine auxotrophy and a mutation in *rpoB*, likely responsible for the rifampicin resistance. Nonsense mutations were detected in PA2735 and PA1939 encoding a DNA methyltransferase and a putative OLD family endonuclease, respectively. Analysis of revertants in these two genes showed that PA2735 is a component of a restriction-modification system, independent of PA1939. Moreover, a 12 kb RPG42 prophage and a novel 108 kb PAPI-1 like integrative conjugative element (ICE) encompassing a mercury resistance operon were identified. The ICE*Pae*1161 was transferred to *Pseudomonas putida* cells, where it integrated in the genome and conferred the mercury resistance.

**Conclusions:**

The high-quality *P. aeruginosa* PAO1161 genome sequence provides a reference for further research including e.g. investigation of horizontal gene transfer or comparative genomics.

The strain was found to carry ICE*Pae*1161, a functional PAPI-1 family integrative conjugative element, containing loci conferring mercury resistance, in the past attributed to the FP2 plasmid of IncP-8 incompatibility group. This indicates that the only known member of IncP-8 is in fact an ICE.

## Background

*Pseudomonas aeruginosa* is a Gram-negative gammaproteobacterium commonly found in various ecological niches and characterized by the ability to survive in unfavourable, frequently changing environmental conditions. This opportunistic pathogen is often a cause of nosocomial infections in immuno-compromised patients. In cystic fibrosis patients *P. aeruginosa* chronically colonizes the lungs and is a major mortality factor [1, 2].

Research on this metabolically versatile bacterium frequently involves sublines or derivatives of *P. aeruginosa* PAO1 strain, originally isolated from a wound of a patient in the Holloway’s laboratory, Melbourne, Australia [3]. Over the years, the strain was shipped to laboratories worldwide and its different attenuated derivatives, including auxotrophic strains and strains with mobile genetic elements were obtained [4]. In 1999 the genome of *P. aeruginosa* PAO1, stored at the University of Washington (PAO1-UW), was sequenced [5], providing a reference for studies on *P. aeruginosa* genomes. Up to October 2019, the Pseudomonas Genome Database, a database devoted to the information on *Pseudomonas* species [6], contained 4660 sequenced *P. aeruginosa* genomes, including 22 PAO1 sublines. Remarkably, sequencing of the PAO1 subline (MPAO1) as well as PAO1-DSM strain stored at the German Collection for Microorganisms and Cell Cultures revealed presence of multiple nucleotide polymorphisms and short insertions-deletions (indels) relative to the reference PAO1-UW [7]. A major feature differing genomes of PAO1 derivatives MPAO1 and PAO1-DSM, is the lack of a large inversion resulting from the homologous recombination between two rRNA operons *rrnA* and *rrnB* [5], which is present in the reference PAO1-UW genome [7]. Despite an asymmetrical positioning of the *dif* region in PAO1-UW, this inversion does not seem to affect chromosome segregation and such large rearrangements might be common among bacteria [8]. Remarkably, recent analyses indicated that sequence variation including single-nucleotide polymorphisms (SNPs), multiple-nucleotide polymorphisms (MNPs) and indels could lead to major variations in e.g. virulence and fitness between strains used in different laboratories [7]. This indicates an ongoing micro- and macro- evolution of bacterial genomes and suggests that sequence diversification in laboratory strains should be taken into consideration in the analysis of phenotypic data [9–12].

In this work we focus on the genome of *P. aeruginosa* PAO1161 strain, a PAO1 derivative requiring leucine for growth on minimal media and selected as defective in its restriction-modification properties (*rmo-*10 mutation) [13]. This strain is described as the host for FP2 plasmid conferring resistance to mercury, the only known member of IncP-8 incompatibility group [14, 15]. The FP2 factor demonstrated the chromosome-mobilizing ability (Cma) and was extensively used in interrupted mating technique for preparation of the genetic map of *P. aeruginosa* chromosome [4, 16].

**Fig. 1:**
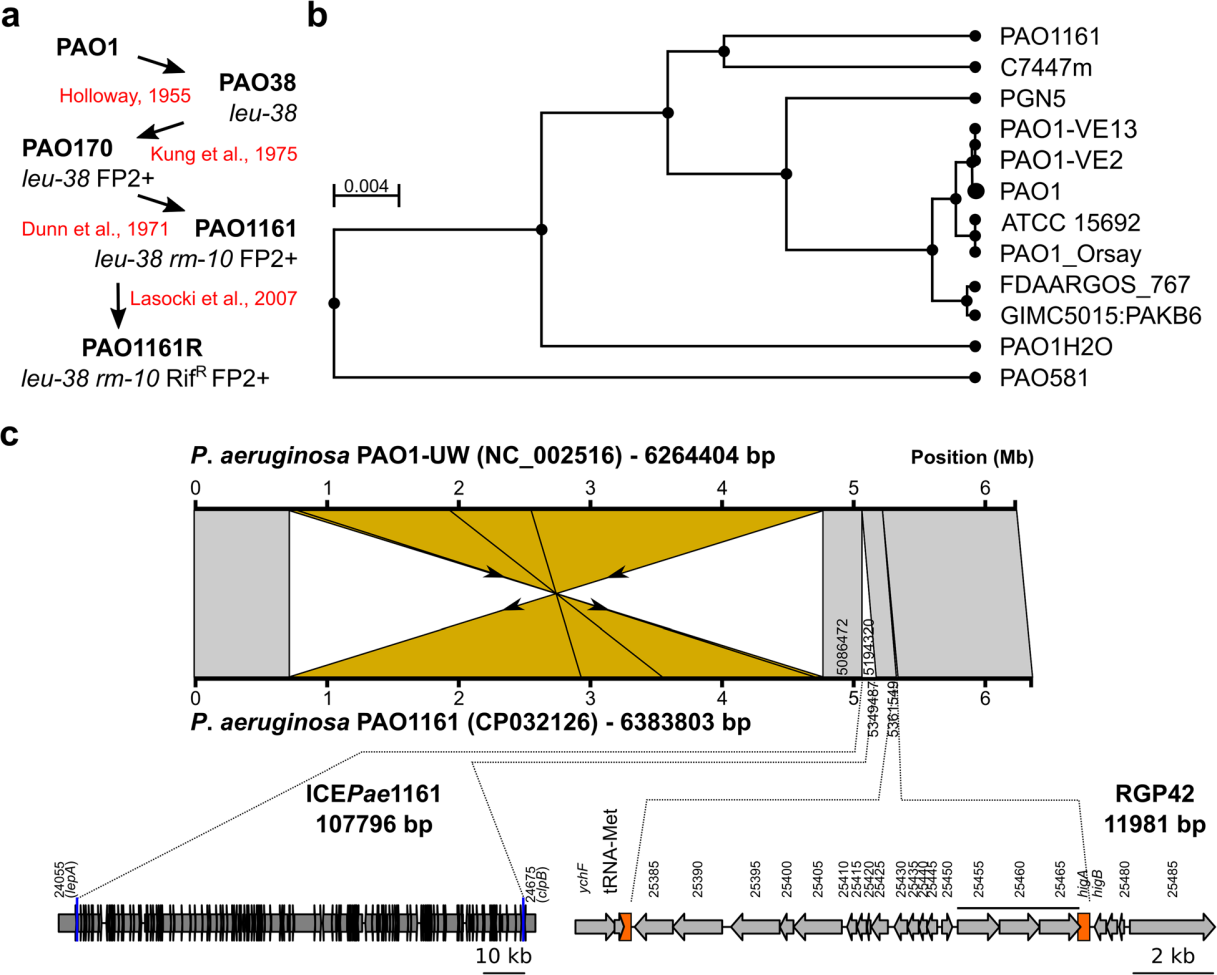
Comparison of *P. aeruginosa* PAO1161 and PAO1-UW genomes. **a** Origin of *P. aeruginosa* PAO1161 strain. **b** A subsection of the phylogenetic tree of *P. aeruginosa* strains deposited at NCBI showing selected strains closely related to PAO1161. The tree was constructed using a Tree View option from the NCBI Web BLAST service [17]. The analysed genomes are listed in Additional file 1: Table S1. **c** Major structural variations between the genomes of the two *P. aeruginosa* strains. Whole genome alignment and synteny visualization was performed with EasyFig [18]. Blocks indicate regions with percentage of nucleotide sequence identity higher than 95%. The inversion between *rrnA* and *rrnB* rRNA operons is coloured in yellow. Bottom panel indicates positions and schematic gene organization of large insertions: ICE*Pae*1161 and RGP42. D3C65_ in the locus IDs of PAO1161 genes was removed for clarity

The PAO1161 derives from the PAO38 *leu*-*38* mutant (Fig. 1a), obtained by treatment of PAO1 with manganese chloride and search for leucine auxotrophs [3]. PAO38 acquired the FP2 element from PAT (*P. aeruginosa* strain 2) [19] to yield strain PAO170 [20]. Following mutagenesis of PAO170 with N-methyl-N′-nitro-N-nitrosoguanidine, PAO1161 was selected as defective in restriction and modification systems (*r*^*−*^*m*^*−*^) on the basis of the altered susceptibility to phage infection [13, 21]. To facilitate the use of PAO1161 in conjugation experiments, a rifampicin resistant clone was obtained [22]. The PAO1161 strain was used in studies on chromosome segregation and gene expression using genome wide approaches [23–25] as well as in other physiological and genetic studies [26–33].

Here we report the genome sequence of *P. aeruginosa* PAO1161 strain. Comparison with PAO1-UW reference sequence revealed the presence of a large number of SNPs, and indels as well as lack of inversion of large genome segment between rRNA genes. Moreover a functional PAPI-1 like integrative conjugative element (ICE), containing a mercury resistance operon was identified in PAO1161 genome, indicating that the FP2 factor is not a plasmid but an ICE (designated ICE*Pae*1161).

## Results and discussion

### Comparison of *P. aeruginosa* PAO1161 genome with PAO1 reference assembly

*P. aeruginosa* PAO1161 genome assembly resulted in a single circular chromosome of 6,383,803 bp. A phylogenetic comparison of PAO1161 genome with other *P. aeruginosa* genomes available in the NCBI database, identified C7447m, a mucoid isolate from a patient with cystic fibrosis [34] as a strain with most similar genome. In the global analysis PAO1161 localized close to the PAO1 containing branch, in agreement with its origin (Fig. 1b). A comparison of PAO1161 genome with the reference PAO1-UW genome (NC_002516) revealed three major structural differences (Fig. 1c). The PAO1161 genome lacks the large inversion between ribosomal RNA operons *rrnA* and *rrnB* observed in PAO1-UW [5] also absent in other PAO1 derivatives like MPAO1 and PAO1-DSM [7]. The correct sequence assembly of the inversion boundaries was confirmed by careful inspection of the coverage of these sections with reads and PCR amplification of the boundaries (data not shown). Remarkably, PAO1161 possesses two large insertions (Fig. 1c). The 107,796 bp insertion in *tRNA*_Lys_ gene between PA4541 (*lepA*) and PA4542 (*clpB*), flanked by 48 bp repeated sequences, displays a significant similarity to PAPI-1 like integrative conjugative elements (see below) [35, 36]. The second 11,981 bp insertion between PA4673.1 (*tRNA*_Met_) and PA4674 (*higA*), flanked by 82 bp repeats, is identical to the prophage-like RGP42 element also identified in MPAO1 and PAO1-DSM [7]. Additionally, PAO1161 lacks a 280 bp fragment containing PA1796.3 and PA1796.4 tRNA genes and has an 107 bp insertion downstream of PA2327.

### Effect of SNPs, MNPs and indels

A comparison of PAO1161 and PAO1-UW genome sequences using Nucdiff [37], followed by a quality check (see Materials and methods) revealed 100 high confidence SNPs, MNPs and short indels. The variants encompassed 52 SNPs, 6 MNPs, 15 deletions and 27 insertions. Of these, 44 were mapping to the intergenic regions in PAO1-UW genome and nine were synonymous (silent) mutations (Additional file 2: Table S2). Three SNPs introduced stop codons leading to the production of truncated proteins (Table 1). These included PA1939 and PA2735 with a predicted role in restriction/ modification. Four of the identified small indels resulted in frame shifts, leading to the expression of proteins with altered C-terminal regions (Table 1). These encompass PA0683 (*hxcY*) encoding a component of the Hxc system, a type II secretion system dedicated to the secretion of alkaline phosphatases LapA and LapB [38, 39]. The effect of 14 indels and 1 SNP is predicted as a shift in start or stop codon of the corresponding gene leading to an extension of the protein product in PAO1161 relative to PAO1-UW (Additional file 3: Table S3).

**Table 1:** SNPs and indels identified in *P. aeruginosa* PAO1161 genome, resulting in expression of truncated proteins. The effect of a mutation is predicted using the PAO1-UW genome as a reference. In case of PA2492 (*mexT*) the nucleotide changes are proposed to alter the start codon and hence the sequence of N-terminal part

Mutation effect	PAO1-UW position	Nucleotide change	AA change	length PAO1/PAO1161	PAO1 gene	PAO1161 ID	Description
stop codon	2,121,203	C → T	W340*	665 /339	PA1939	D3C65_15950	putative ATP-dependent endonuclease of the OLD family
	2,356,682	CC → C	L173*	182 /172	PA2141	D3C65_14865	CinA family protein
	3,097,884	G → A	Q209*	792 / 208	PA2735	D3C65_11725	SAM-dependent DNA methyltransferase
frame-shift	740,419	G → GC	V73	381 / 124	PA0683 (*hxcY*)	D3C65_22610	putative type II secretion system protein
	1,440,623	AA → A	K640	656 /642	PA1327	D3C65_19175	putative protease
	1,835,045	G → GC^a^	S218	249 / 226	PA1685 (*masA*)	D3C65_17305	enolase-phosphatase E-1
	2,807,706	CAGCCGGCC → C	aa1–78/ 35aa	347 / 304	PA2492 (*mexT*)	D3C65_13040	transcriptional regulator

^a^−SNP at this position in PAO1DSM / MPAO-1 [7] but a nucleotide insertion in our study

Except nucleotide changes with a major effect on the corresponding protein products, numerous SNPs and indels resulting in amino acid substitutions or deletions relative to corresponding PAO1-UW proteins were identified (Additional file 4: Table S4). In case of PA2492 (*mexT*) both a deletion (8 bp, Table 1) and a SNP (resulting in F172I change, Additional file 4: Table S4) were observed in PAO1161 relative to PAO1-UW sequence. MexT is a LysR type transcriptional regulator activating expression of the MexEF-OprN multidrug efflux system, extensively studied in the context of quorum sensing signalling and resistance to antimicrobial agents [40, 41]. Mutations in *mexT* are frequently identified in laboratory PAO1 sublines [42].

Interestingly, for 8 proteins the same changes were found in PAO1161 strain and in MPAO1 and / or PAO1-DSM [7] relative to corresponding PAO1-UW proteins (Additional file 4: Table S4). Fifteen changes seem to be PAO1161 strain specific (Additional file 4: Table S4, bolded) as revealed by comparison of the sequences with other members from the corresponding Pseudomonas Ortholog Groups. Summarizing, the identified sequence variations should be considered in analyses of the corresponding proteins using different *P. aeruginosa* strains.

### Functional relevance of the identified sequence variations

*P. aeruginosa* PAO1161 used in this study was a rifampicin resistant clone [22]. Rifampicin binds to a conserved pocket on the β-subunit of RNA polymerase therefore blocking RNA transcript elongation [43]. Resistance to this drug results from mutations in the *rpoB* gene that change the structure of the pocket [43–45]. Our analysis revealed presence of a SNP in *rpoB*, encoding a DNA-directed RNA polymerase subunit beta, resulting in H531L substitution (Additional file 4: Table S4). This amino acid change was frequently observed in spontaneous *P. aeruginosa* Rif^R^ mutants [46], strongly indicating that this SNP confers PAO1161 strain with rifampicin resistance.

**Fig. 2:**
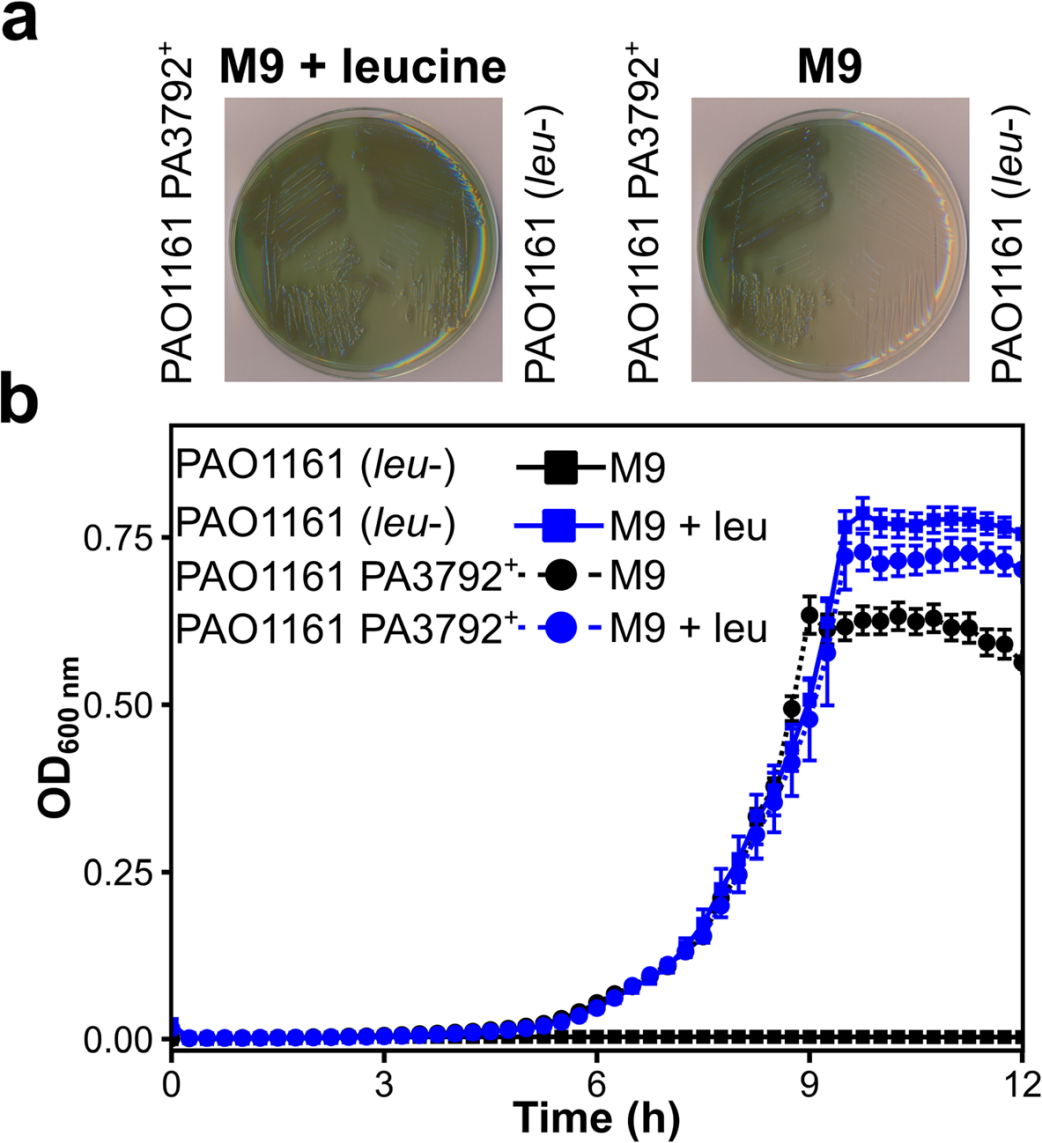
LeuA E108K substitution causes leucine auxotrophy in *P. aeruginosa*. PAO1161 *leuA* allele, carrying the mutation, was replaced with the PAO1 allele to yield strain PAO1161 PA3792^+^ (*leu*+). Growth of PAO1161 (*leu-)* and PAO1161 PA3792^+^ (*leu*+) strains on solid (**a**) and liquid (**b**) minimal medium containing 0.25% citrate with or without 10 μg ml^− 1^ leucine. Data represent mean OD_600nm_ ± SD for 6 biological replicates

PAO1161 strain was derived from the strain PAO38 mutagenized towards leucine auxotrophy (Fig. 1a). Genome sequencing of PAO1161 revealed that this strain possesses a mutation in *leuA*, encoding a putative 2-isopropylmalate synthase, resulting in E108K substitution. Analysis of Pseudomonas Ortholog Group of the *leuA* (POG001874) showed that, the only *P. aeruginosa* strains carrying this mutation are PAO579 [47, 48] and PAO581 [49], two PAO38 derivatives. To validate that this substitution leads to the leucine auxotrophy, we replaced *leuA* allele in PAO1161 by corresponding PAO1 sequence. The replacement fully restored the ability of PAO1161 strain to grow on minimal medium without leucine (Fig. 2), confirming that the E108K substitution in LeuA caused leucine auxotrophy.

### Analysis of PAO1161 revertants in PA1939 and PA2735

*P. aeruginosa* PAO1161 strain was selected as PAO170 defective in its restriction and modification systems (Fig. 1a). Interestingly, two mutations identified in PAO1161 in comparison to PAO1-UW, that resulted in an introduction of premature stop codons mapped to PA2735 gene, recently shown to encode a N6-adenosine DNA methyltransferase acting on a conserved sequence GATC(N)_6_GTC [50, 51], and PA1939, encoding a putative overcoming lysogenization defect (OLD) family nuclease containing an N-terminal ATPase domain and a C-terminal TOPRIM domain [52, 53]. Since OLD proteins can act as exonucleases digesting DNA in the 5′-3′ direction as well as endonucleases acting on supercoiled, circular DNA substrates [53], it was tempting to speculate that PA1939 could play a role in degradation of the foreign DNA in concert with PA2735 acting as methylase.

**Fig. 3:**
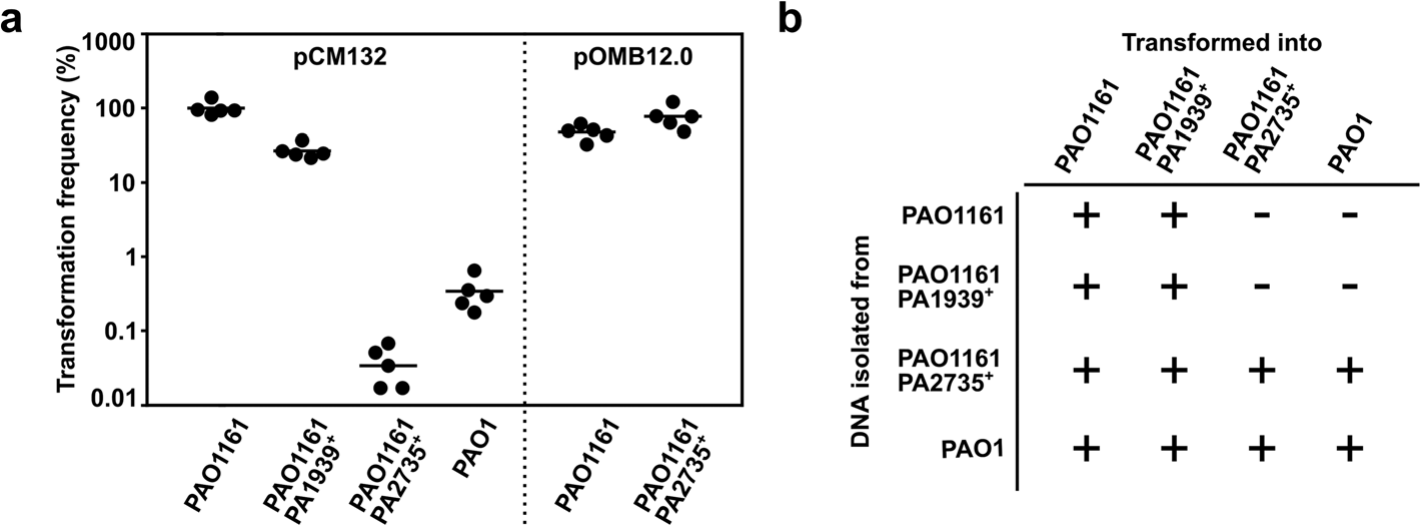
Influence of mutations in PA2735 or PA1939 on plasmid transformation of *P. aeruginosa* cells. **a** Transformation frequency of *P. aeruginosa* strains transformed with plasmids. pCM132 containing 3 sequence motifs recognized by PA2735 and pOMB12 DNA without the motifs were isolated from *E. coli* GM2163 and used to transform the indicated strains. Transformation frequency was calculated as number of transformants relative to the total amount of cells in transformation mixtures. Mean frequency for PAO1161 cells transformed with pCM132 was set to 100%. Lines indicate means and dots indicate results of independent transformations. **b** Influence of the source of plasmid DNA on its ability to transform *P. aeruginosa* strains. pCM132 isolated from the indicated *P. aeruginosa* strains was used for transformation. The experiment was performed twice with identical results. (+) at least 50 colonies on the plates, (−) no colonies

To test the role of PA1939 and its possible cooperation with PA2735, the mutated alleles in PAO1161 genome were replaced by PAO1 wild type alleles to obtain revertants, PAO1161 PA2735^+^ and PAO1161 PA1939^+^. A strain producing the putative endonuclease PA1939 and not producing the methylase PA2735 was obtained and it did not show a growth defect relative to WT (data not shown), indicating that PA2735 methylation is not required for protection against PA1939 action. The obtained PAO1161 revertant strains, producing full length PA1939 or PA2735 were tested for their ability to accept foreign plasmid DNA. We used pCM132, a broad host range plasmid with RK2 replication system [54], carrying three GATC(N)_6_GTC motifs, recognized by PA2735 [50] as well as pOMB12.0, a derivative of broad host range plasmid pBBR1-MSC3 [55], lacking such sequences. DNA was isolated from *E. coli* GM2163 (*dam*^−^, *dcm*^−^), defective in modification systems, and used for transformation of PAO1161 (*r*^−^, *m*^−^), PAO1161 PA2735^+^, or PAO1161 PA1939^+^ and PAO1 (*r*^+^, *m*^+^). A minor (4-fold) reduction of transformation efficiency was observed for PA1939^+^ strain in comparison to PAO1161. Notably, a drastic reduction of transformation frequency in PAO1161 PA2735^+^ and PAO1 strains in comparison with PAO1161 was observed (Fig. 3a), implying that PA2735 participates in specific DNA recognition and degradation, a feature characteristic for type I methyltransferases [56–58], where presence of methyltransferase (HsdM) is required for full activity of the HsdMSR complex. Indeed such reduction in transformation frequency was not observed when a plasmid lacking DNA motifs recognized by PA2735 was used (Fig. 3a, pOMB12.0).

The involvement of PA2735 and PA1939 in DNA modification was also tested. Plasmid DNA isolated from four sets of *P. aeruginosa* transformants was used to transform the four strains. As expected plasmid DNA isolated from PAO1 and PAO1161 PA2735^+^ (with active HsdMSR system) was effective in establishment in all four tested strains. Plasmid DNA isolated from PAO1161 and PAO1161 PA1939^+^ revertant with inactive HsdMSR system and in consequence not modified by methyltransferase is incapable to establish in PAO1 and PAO1161 PA2735^+^ revertant (Fig. 3b). Overall the data indicate a role of PA2735 in plasmid establishment, however the function of PA1939 remains obscure and requires further studies.

### PAO1161 genome contains an ICE conferring resistance to mercury

**Fig. 4:**
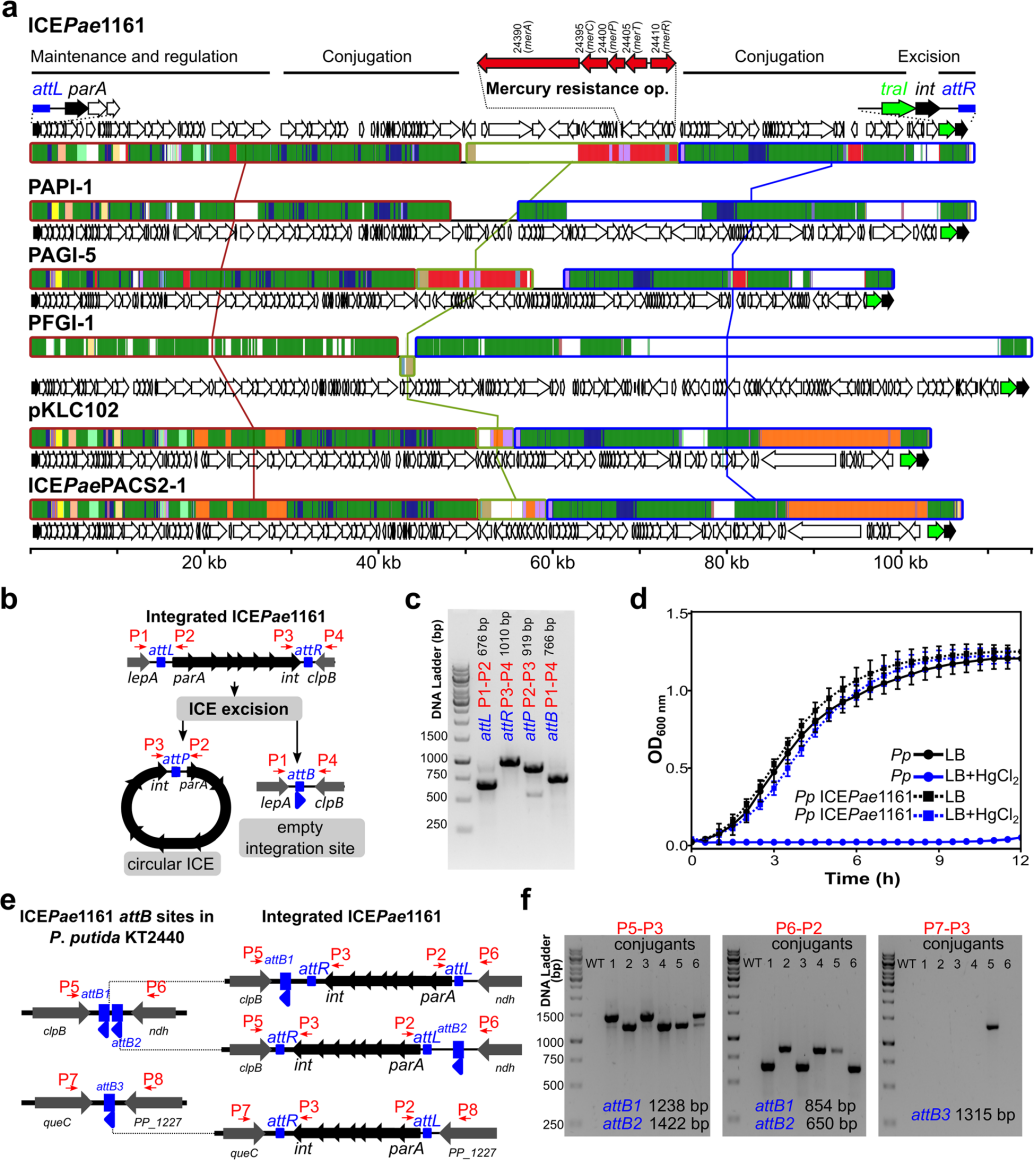
ICE*Pae*1161 identified in *P. aeruginosa* PAO1161 genome is a functional PAPI-1 family integrative and conjugative element conferring resistance to mercury. **a** Comparative genomics of ICE*Pae*1161 and selected PAPI-1 family ICEs. Mauve alignment of ICE*Pae*1161 and PAPI-1 [59], PAGI-5 [60], PFGI-1 [61], pKLC102 [62] and ICE*Pae*PACS2–1 (position 896,693:1002644 of NZ_AAQW01000001) [35] is presented. Three blocks of sequences which are free of genome rearrangements, such as inversions and duplications, are marked with rectangles connected with lines. Green segments indicate sequences conserved in all ICEs (backbone). Regions conserved among subsets of analysed ICEs are color coded. White region is specific only to one analysed ICE. Arrows indicate the location and orientation of coding sequences. Boundary genes and the mercury resistance operon are shown on top. **b** Schematic model of linear and excised (circular) ICE*Pae*1161. **c** PCR analysis of ICE*Pae*1161 excision. PCR was performed with indicated primer pairs flanking *att* sequences using PAO1161 genomic DNA as a template and products were separated on a 1.2% agarose gel followed by DNA visualization using ethidium bromide staining. **d** Growth of *P. putida* KT2440 strain and *P*. *putida* KT2440 ICE*Pae*1161*::aadA* (Sm^R^). Strains were grown in L broth with or without 40 μM HgCl_2_. Data represent mean OD_600nm_ ± SD for 6 clones analysed in 3 biological replicates. **e** Genomic context of three potential ICE*Pae*1161 integration sites (*attB*) in *P. putida* KT2440 genome. The sites were identified based on the presence of 48 bp sequence flanking ICE*Pae*1161 in PAO1161 genome. Blue arrows indicate the orientation of the sequences. Schematic model of ICE*Pae*1161 integration at each *attB* site in orientation corresponding to the one observed in PAO1161 genome. **f** PCR analysis of ICE*Pae*1161 presence in *P. putida* KT2440. Genomic DNA of the wild-type *P. putida* KT2440 (WT) and six independent transconjugants (1–6) was used as a template in PCR with the indicated primer pairs. Products were separated on 1% agarose gel followed by DNA visualization using ethidium bromide staining. Primer binding sites and names are indicated in red (Additional file 6: Table S6)

PAO1161 was described as a strain containing the FP2 plasmid of IncP-8 incompatibility group, which conferred the cells with mercury resistance [19]. Indeed, the strain used in our lab was exceptionally resistant to mercury, growing in L broth supplemented with up to 200 μM HgCl_2_ (data not shown). Surprisingly, during the genome assembly no extra-chromosomal elements could be identified. Instead, an almost 108 kbp insertion in the chromosome, with a putative mercury resistance operon, was found (Fig. 4a). The insertion shows similarities (in sequence and organization/composition of operons flanking the putative integration site) to the PAPI-1 family of integrative conjugative elements (ICEs) abundant in *Pseudomonas* genomes [35, 36, 63]. ICEs are mobile genetic elements, with a modular structure, encoding complete conjugation machinery (usually a type IV secretion system) allowing transfer of their genome to another host. They are reversibly integrated into a host genome and can be passively propagated during bacterial chromosome segregation and cell division [63–65]. PAPI-1 (108 kb, 115 orfs, integrated in *tRNA*_Lys_) was first described in the genome of highly virulent *P. aeruginosa* PA14 strain [59].

The element identified within PAO1161 genome, named ICE*Pae*1161, has an integration site within *tRNA*_Lys_ and PAPI-1 like organization of boundary operons: an operon starting with a gene encoding a putative ParA protein at one end, and an operon encoding a putative relaxase (TraI) and site-specific recombinase (Int) at the other (Fig. 4a). Analysis of gene content, revealed that 102 out of 120 predicted orfs within ICE*Pae*1161, were found in at least one other PAPI-1 like element, whereas orthologs of 41 genes were found in all ICEs analysed (Additional file 5: Table S5).

Integration of ICE into the chromosome as well as its excision is mediated by an ICE encoded site directed recombinase / integrase [66]. Recombination between an attachment site in the chromosome (*attB*) and the corresponding site on a circular ICE (*attP*) leads to integration of the element into the genome, now flanked by identical *attL* and *attR* sequences (Fig. 4b). Excision of the ICE*Pae*1161 and the presence of a circular form was analysed using PCR with primers flanking the *att* sequences (Fig. 4b, c). The analysis confirmed occurrence of the circular ICE in PAO1161 cells (Fig. 4c), indicating that the element can exist in two forms.

To facilitate testing of ICE*Pae*1161 interstrain transfer, we tagged it with a streptomycin resistance cassette (*aadA*). Subsequently, PAO1161 ICE::*aadA* strain (Sm^R^) was used as a donor in mating with *Pseudomonas putida* KT2440 as a recipient in static liquid cultures. The conjugants were selected on M9 plates supplemented with streptomycin, but lacking leucine to block the growth of donor cells. Streptomycin resistant *P*. *putida* clones were obtained with a low efficiency of 2 × 10^− 7^ transconjugants per donor cell.

To confirm that the *P*. *putida* conjugants show also enhanced resistance to mercury, attributed to the presence of *mer* operon within the ICE*Pae*1161, we analysed the growth of transconjugants in medium containing HgCl_2_. The recipient *P. putida* KT2440 cells were unable to grow at a HgCl_2_ concentration higher than 2 μM (data not shown). In contrast, the growth of streptomycin resistant *P*. *putida* transconjugants was not inhibited by the presence of 40 μM HgCl_2_ in the medium, confirming acquisition of mercury resistance (Fig. 4d).

Finally, to confirm that the ICE*Pae*1161 integrated into *P*. *putida* KT2440 chromosome, we searched for putative *attB* sites in its genome using the 48 bp TGGTGGGTCGTGTAGGATTCGAACCTACGACCAATTGGTTAAAAGCCA sequence flanking ICE*Pae*1161 in PAO1161 chromosome. *P. putida* KT2440 genome contains three potential attachment sites designated *attB1*–*3*, with the *attB1* and *attB2* adjacent to each other (Fig. 4e). A PCR analysis, using primer pairs specific to the ICE*Pae*1161/KT2440 chromosome junctions revealed predictably oriented ICE*Pae*1161 in the *P*. *putida* genome (Fig. 4f). Among six individual transconjugants, the specific PCR products were observed preferentially for ICE integrated in one of adjacent sites, *attB1* or *attB2* and one clone demonstrating the ICE integration in *attB3* (Fig. 4f). Overall, these data confirm the ability of the ICE*Pae*1161 identified in the PAO1161 genome to transfer to another host, integration into chromosome at a specific site and conferring mercury resistance.

## Conclusions

In this work we show that *P. aeruginosa* PAO1161 strain carries a PAPI-1 family integrative conjugative element capable of excision, transfer and integration in the genome of another *Pseudomonas* species. The ICE*Pae*1161 contains loci conferring mercury resistance, in the past attributed to the FP2 plasmid of IncP-8 incompatibility group.

The genome sequence of *P. aeruginosa* PAO1161 strain, a derivative of the reference PAO1 strain, was compared with the reference PAO1 sequence. The data indicated a number of sequence variants, thus sequencing, providing insight into genotypes of laboratory strains, is highly recommended to help in the interpretation of phenotypes observed in different laboratories.

## Methods

### Strains and growth conditions

Bacterial strains, plasmids and oligonucleotides used in this work are listed in Additional file 6: Table S6. *P. aeruginosa* PAO1161 (*leu*^−^, *r*^−^, *m*^−^) was provided by B. M. Holloway (Monash University, Clayton, Victoria, Australia). *Escherichia coli* strain DH5α was used for plasmid manipulations and S17–1 was used to mate pAKE600 [67] derivatives into *P. aeruginosa*. Standard DNA manipulations were performed as described [68]. Templates for PCRs were prepared by boiling the cells pelleted from overnight cultures and resuspended in water.

Bacteria were grown in L broth [69] at 37 °C or on L agar (L broth with 1.5% w/v agar) supplemented with appropriate antibiotics. For selection of *E. coli* strains 150 μg ml^− 1^ benzylpenicillin sodium salt in liquid medium, 300 μg ml^− 1^ for solid media, 10 μg ml^− 1^ tetracycline, 50 μg ml^− 1^ kanamycin or 30 μg ml^− 1^ streptomycin was used. *P. aeruginosa* and *P. putida* strains were selected by addition of 300 μg ml^− 1^ carbenicillin, 300 μg ml^− 1^ rifampicin, 100 μg ml^− 1^ tetracycline, 500 μg ml^− 1^ kanamycine or 150 μg ml^− 1^ streptomycin to the medium. Growth analysis was performed in M9 minimal medium [68] with 0.25% citrate or 0.1% glucose supplemented with 10 μg ml^− 1^ leucine or 40 μM HgCl_2_ as indicated. Bacterial growth in 96-well plates was monitored by measurements of optical density at 600 nm (OD_600_) using a Varioskan Lux Multimode Microplate Reader and SkanIt RE 5.0 software (Thermo Fisher Scientific).

### Plasmids and strains construction

To construct prototrophic *P. aeruginosa* PAO1161 strain, which produces LeuA without the E108K substitution, the suicide plasmid pKAB607 was constructed. Sequences flanking the site in PAO1161 chromosome were amplified using primer pairs 1#, 2# and 3#, 4#, respectively. Primers 2# and 3# contained sequence lacking the mutation observed in PAO1161 genome relative to PAO1-UW and introduced an AflII site, allowing selection of the allele. The obtained PCR fragments were digested with EcoRI, AflII and AflII, BamHI, respectively, and both fragments were ligated with EcoRI, BamHI digested pAKE600 (to yield pKAB607). *E. coli* S17–1 strain, carrying pKAB607, was used in the allele exchange procedure performed as described before [22]. Presence of modified allele was verified by AflII digestion of PCR amplified chromosomal *leuA*.

To construct *P. aeruginosa* PAO1161 strain, which lacks the internal stop codon in PA1939, the suicide plasmid pKAB617 was constructed. Two regions of PA1939 were amplified using primer pairs 9#, 10# and 11#, 12#, respectively. Primers 10# and 11# introduced an NruI site without changes in the coding sequence. The obtained PCR fragments were digested with HindIII, NruI and NruI, BamHI, respectively, and both fragments were ligated with HindIII, BamHI digested pAKE600 to yield pKAB617. *E. coli* S17–1 strain, carrying pKAB617, was used in the allele exchange procedure [22]. Presence of the modified allele was verified by NruI digestion of PCR amplified chromosomal PA1939.

To construct *P. aeruginosa* PAO1161 strain producing full length PA2735, the suicide plasmid pKAB618 was constructed. Two fragments of PA2735 were amplified using primer pairs 13#, 14# and 15#, 16#, respectively. Primers 14# and 15# introduced an AseI site without the change in the coding sequence. The obtained PCR fragments were digested with HindIII, AseI and AseI, BamHI, respectively, and both fragments were ligated with HindIII, BamHI digested pAKE600 [70] to yield pKAB618. *E. coli* S17–1 strain, carrying pKAB618, was used in the allele exchange procedure [22]. Presence of modified allele was verified by AseI digestion of PCR amplified chromosomal PA2735.

*P. aeruginosa* PAO1161 ICE::*aadA* (Sm^R^) strain was constructed by insertion of a streptomycin resistance cassette (*aadA*) between orfs D3C65_24365 and D3C65_24370 within ICE*Pae*1161 (Additional file 5: Table S5). To this end, the fragments flanking the region of insertion were amplified from PAO1161 genomic DNA using primer pairs 5#, 6# and 7#, 8#, respectively. The obtained PCR fragments were digested using MunI, HindIII and HindIII, BamHI, respectively, and the mixture was ligated with EcoRI, BamHI digested pAKE600 to yield pKAB608. The Sm^R^ cassette, excised with HindIII from pHP45Ω [71], was ligated with HindIII digested pKAB608 to yield pKAB609. *E. coli* S17–1 (pKAB609) strain was used as a donor in the allele exchange procedure with strain PAO1161 Rif ^R^ [22]. Integration of Sm^R^ cassette was confirmed by PCR.

### Genome sequencing

PAO1161 genome assembly was performed by combining long reads obtained using MinION (Oxford Nanopore Technologies, Oxford, UK) and Illumina reads used previously as the input sample in chromatin immunoprecipitation-sequencing experiments [23]. The Illumina data encompassed 23,380,926 reads (4,676,185,200 nt). Obtained reads were quality-filtered using FastX toolkit (http://hannonlab.cshl.edu/fastx_toolkit/) and residual Illumina adapters were removed using Cutadapt (https://github.com/marcelm/cutadapt) [72]. A subsample (7000 000 reads) was used in draft genome assembly using Spades v3.11.1 (http://cab.spbu.ru/software/spades/) to estimate the size of PAO1161 genome. The long reads were generated using MinION. Genomic DNA was sheared into 20 kb fragments using Covaris gTube (*Covaris*, Ltd., Brighton, United Kingdom) and the library was prepared using an ONT 1D ligation sequencing kit (SQK-LSK108) with the native barcoding expansion kit (EXP-NBD103). Nanopore sequencing was performed using the NC_48 h_Sequencing_Run_FLO-MIN106_SQK-LSK108 protocol, R9.4.1 MinION flowcell and a MinION MkIB instrument. Raw Nanopore data were basecalled using Albacore v2.3.1 (Oxford Nanopore Technologies, Oxford, UK). Reads were quality-filtered using NanoFilt [73] and Porechop (https://github.com/rrwick/Porechop) was used for Nanopore adapter removal. Overall, MinION sequencing yielded 161,877 reads (2,160,720,766 nt), with a median read length of 12,489 nucleotides. Long Nanopore reads were assembled in a hybrid mode with the Illumina data using Unicycler v.0.4.6 [70]. Genome assembly consisted of single circular molecule of the size 6,383,803 bp. Errors identified in the assembly were verified by a PCR amplification of DNA fragments, followed by Sanger sequencing on an ABI3730xl Genetic Analyzer (Life Technologies, USA) using BigDye Terminator Mix v. 3.1 chemistry (Life Technologies, USA) followed by a manual correction of the genome sequence using Seqman software (DNA Star, USA). Out of 9 detected problems 4 conflicts were identified as SNPs and five indels were identified and polished respectively. Final genome consensus sequence resulted in one circular replicon of 6,383,803 bp. The genome assembly completeness was assessed using Busco software [74].

The assembled genome was annotated using the NCBI Prokaryotic Genome Annotation Pipeline [75]. The nucleotide sequence has been deposited in NCBI Nucleotide database (accession number CP032126).

### Genome analysis

Genome synteny between PAO1161 and PAO1-UW was visualized using EasyFig [18]. Structural variations, SNPs, insertions and deletion between the PAO1161 and PAO1-UW (NC_002516) sequences were identified using Nucdiff [37] yielding 251 variations. Since a direct sequence comparison does not provide information about the quality of predicted variants, the outcome can be greatly affected by errors during genome sequence consensus calling, caused for instance by mapping of reads derived from highly similar sequences to another, very similar, parts of the genome. To perform a sequence quality control, short reads were mapped to the PAO1161 genome using Bowtie v2.3.4.2 [76] followed by a verification of the quality of the assembly in regions differentiating PAO1161 and PAO1-UW genomes. Percentage of a given base, relative to bases from all reads, at positions in PAO1161 genome corresponding to the identified SNPs, MNPs and short insertions was analysed using bam-readcount (https://github.com/genome/bam-readcount). Regions with short deletions were inspected using Integrative Genomics Viewer v2.4.9 [77]. The variants present in more than 80% of the reads with an average mapping quality > 20, were considered as homozygous. The remaining (heterozygous) variants were mostly SNPs (149/151) located in coding sequences of genes belonging to Pseudomonas Ortholog Groups (POG) possessing multiple members in the same strain (multiple orthologs within PAO1 genome) [6]. The effect of SNP/MNP and indels was predicted using snpEff [78].

### Transformation frequency

Competent cells of *P. aeruginosa* were prepared with the magnesium chloride based method [79]. pCM132 or pOMB12.0 were propagated and isolated from *E. coli* GM2163 strains (*dam*^−^ and *dcm*^−^) and 500 ng was used for transformation of *P. aeruginosa* strains. pCM132 plasmid DNA was also isolated from obtained transformants of *P. aeruginosa* strains and used again in transformation of *P. aeruginosa* strains to compare the efficiency of transformation. Transformation frequency was calculated as number of transformants relative to the total amount of cells in transformation mixtures estimated by plating and counting of colony forming units.

### ICE analysis and conjugal transfer

Comparative analysis of ICEs and gene ortholog prediction was performed using Mauve v2015–02–13 [80]. GenoplotR was used to visualize gene distribution [81]. ICE*Pae*1161 transfer from *P. aeruginosa* PAO1161 ICE*::aadA* (Sm^R^) to *P. putida* KT2440 was performed by growing the strains in L broth overnight at 37 °C, harvesting the cells, resuspending in the same amount of medium and mixing in 1:2 (donor: recipient) ratio in 1.5 ml tubes. The mixtures were incubated for 2 h at 37 °C with shaking (300 rpm) and then 3 h without shaking, followed by centrifugation and resuspension of the cells in the same volume of 0.9% NaCl. To estimate the efficiency of ICE*Pae*1161 transfer a suspension was serially diluted in 0.9% NaCl and aliquots were spotted onto M9 minimal medium agar plates with 0.1% glucose and 150 μg ml^− 1^ streptomycin without leucine. Lack of leucine allowed growth of *P. putida* KT2440 transconjugants and counter selected PAO1161 *leu*^*−*^ donor cells. Donor or recipient strain cultures treated in the same way were used to establish the titer of donor and recipient cells in the conjugation mixture. The transfer frequency was calculated as the number of transconjugants per donor cell.

## Supplementary information

**Additional file 1: Table S1.** *P. aeruginosa* genomes used to construct phylogenetic tree presented in Fig. 1b.

**Additional file 2: Table S2.** Synonymous and intergenic SNPs/MNPs and indels identified in PAO1161 / PAO1-UW genome comparison.

**Additional file 3: Table S3.** SNPs and indels identified in *P. aeruginosa* PAO1161 genome changing the reading frames of genes. The effect of a mutation is predicted using the PAO1-UW genome as a reference. The sequences of PAO1161 proteins were compared with sequences from corresponding Pseudomonas Ortholog Group (POG) for PAO1 proteins.

**Additional file 4:** **Table S4.** SNPs and indels identified in *P. aeruginosa* PAO1161 genome resulting in amino acid changes relative to corresponding PAO1-UW proteins. PAO1161 strain specific changes, identified by comparison of PAO1161 proteins with sequences in Pseudomonas Ortholog Groups encompassing corresponding PAO1 proteins, are indicated by bolded gene names.

**Additional file 5: Table S5.** Gene content of ICE*Pae*1161. Orthologs from other ICEs were predicted using Mauve assuming 70% of identity and at least 70% of the sequence coverage.

**Additional file 6: Table S6.** Strains, plasmids and primers used in this study.

## Data Availability

The data generated and analyzed in this study are included in the article (and its Additional files). The nucleotide sequence has been deposited in the NCBI Nucleotide database and is accessible under accession number CP032126.

## Abbreviations

*att*: attachment site
bp: base pair
ICE: integrative conjugative element
indels: insertions-deletions
kbp: kilo base pair
M9: minimal medium
MNP: multiple nucleotide polymorphism
NCBI: National Center for Biotechnology Information
nm: nanometer
nt: nucleotide
OD: optical density
orfs: open reading frames
PCR: polymerase chain reaction
Rif^R^: rifampicin resistance
rRNA: large subunit ribosomal ribonucleic acid
Sm^R^: streptomycin resistance cassette
SNP: single nucleotide polymorphism
